# Premature Hair Graying and Its Associated Factors Among Medical Students and Resident Physicians at Imam Abdulrahman Bin Faisal University

**DOI:** 10.3390/healthcare13101185

**Published:** 2025-05-19

**Authors:** Inaam B. Aldamanhori, Nada J. Alghamdi, Sadan M. Alharbi, Shadan A. Aljarri, Haya A. AlHemli, Moataza M. Abdel Wahab

**Affiliations:** 1 Department of Dermatology, College of Medicine, Imam Abdulrahman Bin Faisal University, Dammam 31441, Saudi Arabia; 2 Department of Dermatology, Armed Forces Hospital, Dhahran 34641, Saudi Arabia; 3 College of Medicine, Imam Abdulrahman Bin Faisal University, Dammam 31441, Saudi Arabia; 4 Family and Community Medicine Department, College of Medicine, Imam Abdulrahman Bin Faisal University, Dammam 31441, Saudi Arabia

**Keywords:** premature graying of hair, early canities, Saudi Arabia, perceived stress level

## Abstract

**Background:** Premature hair graying (PHG) is one of the most prevalent conditions affecting individuals worldwide. It has been recognized as an important cause of low self-esteem, with a significant physical and social impact. This study investigated the factors associated with PHG by comparing medical students and resident physicians to other non-medical majors at Imam Abdulrahman Bin Faisal University (IAU). **Methods:** This is a comparative cross-sectional study conducted on resident physicians and students from 10 different colleges of IAU, Eastern Province, Saudi Arabia. This study evaluated the characteristics of PHG and its associated risk factors using an online distributed questionnaire. **Results:** A total of 2644 students and resident physicians were included in this study, with 45.6% coming from medical colleges. The findings show that the premature graying of hair was higher among obese and overweight individuals and those with a combined deficiency of vitamins B12 and vitamin D. Other factors associated with PHG included a family history of PHG, a lack of exercise, smoking, and allergic rhinitis. Gray hair onset before 25 years old was higher among those with high stress levels (95%) than those with moderate (90%) and or mild (86%) stress levels (*p* = 0.029). **Conclusions:** In our study, higher stress levels were related to an earlier age of PHG onset, and there was no difference between medical and non-medical students in PHG. Recommendations for future research include randomized clinical trials and larger cohort studies regarding the characteristics of PHG in the Middle East and those with Arabic ethnicity and assessing the medications that induce possible hair repigmentation.

## 1. Introduction

Premature hair graying (PHG) is one of the most prevalent conditions affecting individuals worldwide, with a prevalence of 27.3% [1]. It has been recognized as an important cause of low self-esteem, having a significant physical–social impact [2]. Although the definition of premature hair graying (PHG) remains consistent, its operationalization in research may vary across racial and ethnic groups, corresponding to differences in the typical age of onset; PHG is generally considered to occur before the age of 20 in white people, before 25 in people from Asia, and before 30 in people from Africa [2,3]. Previous studies among individuals of Saudi descent considered the appearance of gray hair before the age of 30 to be premature [4,5]. The exact pathophysiological mechanism of PHG remains incompletely understood. However, it revolves around the role of antioxidants in the growth of hair follicles and an increase in oxidative stress, which ultimately damages melanocytes and leads to a loss of pigmentation [1,2]. Multiple factors can predispose patients to this increase in oxidative stress, such as emotional stress, vitamin B12 deficiency, thyroid hormone deficiency, smoking, genetic predisposition, or autoimmune conditions such as vitiligo. Moreover, PHG can be inherited in an autosomal dominant inheritance pattern [3,6,7,8].

Multiple cross-sectional studies from 2013 to 2023 in India, Nepal, Pakistan, Turkey, Korea, Indonesia, Saudi Arabia, and Egypt have assessed the prevalence of PHG and its risk factors [1,6,7,8,9,10,11,12,13,14,15]. There has been a variation in the results of these studies owing to the definition of PHG, which relies on ethnicity. In the Middle East, there is little evidence regarding PHG as a reference age group. However, recent studies conducted in Saudi Arabia and Egypt have assessed PHG with a cutoff of 30 years as an inclusion criterion [4,5,9].

The reported prevalence of PHG among medical students ranged from 31.2% to 41.4% [16,17,18,19], compared to 27.3% in the general population [1]. A study conducted among medical students in Indonesia reported a prevalence of 71.2% [20]. The risk factors that were reported to be significantly associated with PHG among the studies were family history, obesity, smoking, educational status, hair loss, and stress [7,12,14]. Hypothyroidism and nutritional deficiencies including ferritin, vitamin B12, vitamin D, and calcium also had a significant association with PHG [7,8,11]. Other factors included a preference for a vegetarian diet, a low body mass index (BMI) (<18.5), a history of atopy, a sedentary lifestyle, the application of oils on the scalp, and coronary artery disease [6,7,8,11,21].

Due to the demanding academic and clinical training that medical students and resident physicians undergo, they are particularly vulnerable to high levels of stress, which may contribute to early aging markers, such as PHG [9]. Hence, in this study, we aimed to investigate the factors associated with PHG by comparing medical students and resident physicians to other non-medical majors at Imam Abdulrahman Bin Faisal University (IAU) in the Eastern Province, Kingdom of Saudi Arabia.

## 2. Material and Methods

### 2.1. Study Design, Setting, and Ethics

This comparative cross-sectional study included undergraduate students from ten colleges, as well as resident physicians (postgraduate students), at Imam Abdulrahman Bin Faisal University in Al-Khobar, Eastern Province, Saudi Arabia. The target group consisted of medical students and residents, and the comparative group consisted of students from non-medical field colleges of the same university, including the colleges of science, education, art, computer science, business administration, architecture and planning, engineering, sharia and law, and the college of applied studies and community service. The study obtained ethical approval from the university’s Institutional Review Board (IRB), with an approval date of 10 August 2023 and IRB number IRB-2023-01-315. The study complied with the ethical standards of research ethics involving human subjects (Declaration of Helsinki) [22]. Data collection was performed from August 2023 to December 2023.

### 2.2. Participants

The inclusion criteria were students enrolled in the university and aged 18–30 years. Participants with a hypopigmentary disorder were excluded. All the medical students were approached at different levels. The comparative non-medical field group was selected through stratified random sampling according to theoretical and practical simple random sampling with proportional allocation. Participants were recruited via student groups on WhatsApp and Telegram, which are widely used instant messaging platforms.

### 2.3. Data Collection Tool and Processes

Data were collected using a self-administered online questionnaire developed in Arabic by the research team through the QuestionPro platform. A pilot study was conducted to ensure the clarity and appropriateness of the survey items prior to distribution. The final questionnaire comprised three structured sections. The first section collected sociodemographic information, including age, sex, marital status, financial income, educational level, college affiliation, and academic performance. The second section focused on premature hair graying (PHG) and psychological stress, including the self-reported presence and extent of hair graying, age at onset, and the prior use of hair oils or minoxidil. Psychological stress was assessed using the validated Arabic version of the Perceived Stress Scale (PSS-10) with a Cronbach’s alpha of 0.67 [23]. The third section addressed potential PHG-associated factors, including self-reported medical conditions, vitamin and mineral deficiencies, family history of PHG, dietary habits, physical activity (measured by average daily steps), and smoking. For participants with a history of smoking, additional information on lifetime cigarette exposure was obtained. An optional item allowed participants to request follow-up contact via phone or email for psychological support or stress evaluation.

### 2.4. Variables

This study included a comprehensive set of variables categorized into sociodemographic characteristics, variables related to premature hair graying (PHG) and psychological stress, and potential PHG-associated factors. Sociodemographic variables comprised sex (male, female), age (mean and standard deviation), marital status (married, single, widowed/divorced), financial income (not enough, enough, more than enough), educational level (undergraduate, postgraduate), college affiliation (medicine, science, art, education, computer science, business administration, engineering, architecture and planning, applied studies and community service, and sharia and law), and academic performance, which was categorized based on the participants’ self-reported grade point average (GPA) as good, very good, or excellent. Variables related to PHG and psychological stress included the presence of hair graying (yes/no), the estimated number of gray hairs (<10, 10–100, >100), the age of onset of gray hair (before 25 years, between 25 and <30 years, or 30 years and above), and the use of hair oils or minoxidil prior to the appearance of gray hair (yes/no). Psychological stress was assessed using the validated Arabic version of the Perceived Stress Scale (PSS-10), which consists of 10 items. Each item of the PSS-10 is rated on a 5-point Likert scale (0 = Never, 1 = Almost Never, 2 = Sometimes, 3 = Fairly Often, 4 = Very Often). The total score, ranging from 0 to 40, is obtained by summing all items and is categorized as follows: 0–13 = low stress, 14–26 = moderate stress, and 27–40 = high stress. PHG-associated factors included the presence of specific medical conditions (yes/no), such as depression, hypothyroidism, coronary artery disease, atopic dermatitis, bronchial asthma, and allergic rhinitis. Nutritional deficiencies were assessed based on self-reported serum measurements for vitamin B12, vitamin D, combined vitamin D and B12, calcium, and iron. Participants were asked whether they had been tested for these nutrients and, if so, whether deficiencies had been diagnosed based on their serum measurements (Yes, only a deficiency in [vitamin/mineral]/Yes, a deficiency in both/No deficiency/and Test was not performed.) Additional variables encompassed a family history of PHG and lifestyle-related factors, including the participants’ diet type (vegetarian, mostly meat, or mixed) [7], average number of daily steps (<3000; 3000–6000; 6001–10,000; >10,000) [24], and smoking status (current, past, occasional, second-hand exposure, or non-smoker) [25]. For participants who reported a history of smoking, the lifetime number of cigarettes smoked was categorized as fewer than 100 or more than 100.

### 2.5. Sample Size and Power Analysis

Sample size was calculated using an online sample size calculator (epi info v5.5.10) [26], assuming that the percentage of gray hair in the general population is 27.3% [27]. At a 95% confidence interval (CI) and 80% statistical power, to detect a significant odds ratio of 1.5, the minimum required sample size was 934, in which 467 participants were equally stratified from the target group and the comparative groups.

### 2.6. Statistical Analysis

IBM SPSS Statistics for Windows, version 26.0, was used for the data entry and analysis. Statistical significance was set at a *p* value < 0.05. Bivariate analyses were conducted using premature hair graying (PHG) as a binary variable, defined as the presence of graying before the age of 30. Categorical data were described as frequencies and percentages, while means and standard deviations were used to describe quantitative data. The chi-square test was used to evaluate the association between variables. Logistic regression models were used to identify the independent factors associated with PHG (appeared before the age of 30 years) and to control for confounders.

## 3. Results

Table 1 presents the characteristics of the study participants. In this study, 2644 students were included, with the majority (95%) being undergraduates. Almost half of the students (45.6%) were from medical colleges. Female participants accounted for 64%. Most patients were single (90.4%). The highest percentage (62.7%) of these students had a sufficient financial status. Almost half of the students’ academic performances were very good.

Table 2 shows the characteristics of PHG among university students from the medical and non-medical fields. This shows that 39.1% observed the graying of hair. Almost 70% of them noticed less than 10 gray hairs, and 6.7% noticed more than 100 gray hairs. Regarding the onset of gray hair, 90% of them had it before the age of 25 and 5.9% of them had it between the ages of 25 and 30. Almost 30% of them mentioned the use of hair oils and 13% mentioned the use of minoxidil prior to the onset of hair graying. Among those who had graying hair, the level of stress was moderate in 65.3% (95% CI = [62.4, 68.3]) and high in 20% (95% CI = [17.6, 22.6]), which is higher than that previously reported (12.7%) among Saudi university students [27].

**Figure 1:**
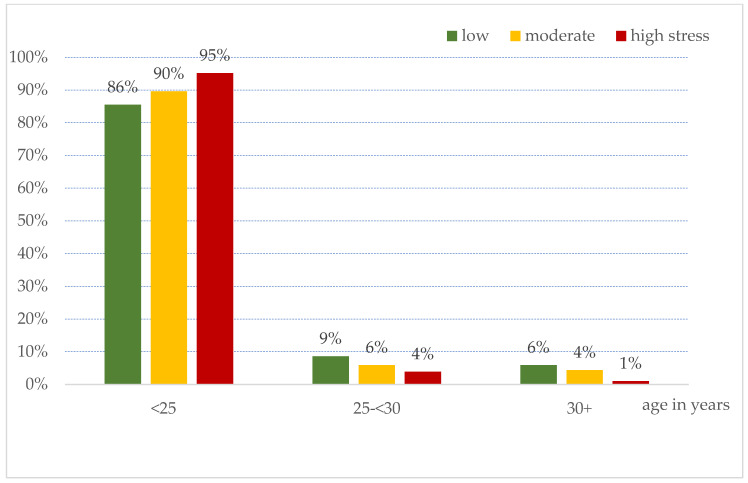
Association between stress levels and age at onset of grey hair.

Figure 1 shows that the onset of gray hair before 25 years old was higher among those with high stress levels (95%) than those with moderate (90%) or mild (86%) stress levels (*p* = 0.029). However, no association was found between the number of gray hairs and stress level.

Participants’ characteristics were compared between students with and without PHG, as illustrated in Table 3. PHG was higher among obese and overweight individuals and those with a combined deficiency of vitamin B12 and vitamin D. Among the triad of atopy, allergic rhinitis demonstrated a statistically significant association with PHG (*p* = 0.018); other factors associated with PHG included a family history of PHG, a lack of exercise represented by a low number of daily steps, smoking, and smoking a high number of cigarettes. Sex, marital status, diet type, financial status, and calcium and iron deficiencies were not significantly associated with PHG. All the values are listed in Table 3.

After entering the studied variables into a logistic regression model, the independent factors associated with PHG included a family history of PHG, a lack of or low exercise, smoking, overweight, and obesity. All the values are listed in Table 4.

Furthermore, by creating a model for only those who were known to have vitamin deficiencies (diagnosed by previous laboratory results) and controlling for the above factors, both vitamin D and B12 were a positive factor associated with PHG, with an adjusted odds ratio = 1.48 (95%CI [1.06, 2.09]), and also those diagnosed with depression (OR = 1.5, 95%CI [1.03, 2.2]).

## 4. Discussion

PHG is the presence of gray hair before the age of 20–30 years, depending on ethnicity [1,3]. In Saudi Arabia, where our study was conducted, the age of PHG was considered to be 30 years and below, according to previous studies conducted in Saudi Arabia [4,5].

The mechanism of action of PHG remains uncertain, but it is likely caused by oxidative stress in the growing hair follicle, which leads to melanocyte damage and a loss of pigment within the hair follicle [2,10]. The multiple factors mentioned earlier contribute to an increased risk of PHG. Of these, stress is a significant contributing factor [12].

Our study was conducted to investigate the factors associated with PHG by comparing medical students and resident physicians below the age of 30 to other non-medical majors at IAU.

We found that the percentage of students with PHG was 39.1% among students at IAU, which was higher than the 28.2% found in a previous study reported by Kansal et al. on university students at a university in Mysuru [28], and close to another study conducted by Rehab Almutairi in Saudi Arabia at another university in Al-Hassa, which found that 42.5% of students had PHG [4]. Moreover, no difference was observed between medical and non-medical specialties (*p* = 0.891) or between males and females (*p* = 0.478).

In our study, the age at PHG onset was associated with higher stress levels. Gray hair onset before 25 years of age was higher among those with high stress levels than among those with moderate and mild stress levels (*p* = 0.029). Recently, Zhang et al. reported how stress may cause PHG. They found that stress induces noradrenaline release from sympathetic nerves, which in turn depletes the melanocyte stem cell niche that provides pigments to hair follicles [29]. It was found that external factors such as psychological stress can also contribute to oxidative stress outside the melanocytes of hair follicles, further challenging the antioxidant capacity of melanocytes and leading to increased damage in aging hair follicles [3]. However, there was no association between the level of stress and the severity of PGH, as illustrated by the number of gray hairs.

Interestingly, the higher the BMI, the higher the prevalence of hair graying before the age of 30 years. In our study, 45.3% of overweight students had PHG and 44.8% of obese students had PHG, as opposed to 32.9% of underweight students and 34.5% of students with a normal BMI and PHG. This result supports previous data reported by Kansal et al. in a study conducted in Mysuru [28], which confirmed the proportional relationship between BMI and PHG. These results support the idea that obesity may reduce melanin production due to the common occurrence of leptin resistance in obese individuals, which in turn increases the levels of substances that counteract melanocyte-stimulating hormone [14,30].

It was found that almost 30% of the students with PHG mentioned the use of hair oils and 13% mentioned the use of minoxidil prior to the onset of hair graying. Further studies are required to investigate the association between their use and PHG levels.

In our study, we also found that students with combined vitamin D and B12 deficiency, confirmed by a history of serum measurement, had a higher prevalence of PHG (43.9%) than those with either vitamin D or B12 deficiency. A study by Chakrabaty et al. in Bengaluru found that serum levels of vitamin B12 were significantly lower in people with PHG than in controls, with no association between vitamin D3 levels and PHG [31]. Hair follicle cells are known for their rapid division. However, the growth of these cells relies on DNA synthesis, which in turn requires an adequate supply of vitamin B12 [32]. Vitamin B12 helps stabilize the initial growth phase of hair follicles [33].

Studying the association between PHG and family history, we found that there is a significant relation between them, where 61.7% of patients with PHG have a family history of PHG. This study confirms the previously reported association, as in studies conducted in India and Indonesia [17,20,28,34].

Our study also revealed that approximately half of the students (48.3%) who were current smokers had PHG, and those who smoked a higher number of cigarettes per day had a higher percentage of PHG. Smoking is a major contributor to oxidative stress and plays an important role in the development of PHG. This finding was confirmed in many studies, such as those conducted by Shin et al., Kansal et al., Bhramaramba et al., and Zayed et al. [14,28,34,35].

Interestingly, allergic rhinitis is also a risk factor for PHG, as individuals with allergic rhinitis were found to have a higher prevalence of PHG (43.7%). The relationship between atopy and PHG remains unclear, but a study by Acer et al. confirmed this association [7]. Further studies are warranted.

A sedentary lifestyle not only affects the coronary arteries, but also affects PHG. This accelerates the aging process in all aspects. We found that people with daily steps of < 3000 had a higher prevalence of PHG at around 40%, whereas those who walked more than 10,000 steps per day had a prevalence of PHG of 28.2%. Chakrabaty et al. confirmed this association [31].

From the factors studied, it was found that sex, marital status, type of diet, financial status, calcium deficiency, and iron deficiency were not statistically significantly associated with PHG.

This study had some limitations. This was survey-based research, and the serum levels of vitamins and iron were not measured. Moreover, it was lacking a clinical examination to evaluate PHG severity.

## 5. Conclusions

In our study, PHG was noticed in almost 37% of students, with no difference between medical and non-medical students. Higher stress levels were related to earlier PHG onset. Recommendations for future research include randomized clinical trials and larger cohort studies on the characteristics of PHG in the Middle East region and among individuals of Arabic ethnicity, as well as assessments of medications that may induce hair repigmentation. Furthermore, additional research is needed to clarify the relationship between PHG and allergic rhinitis.

## Figures and Tables

**Table 1:** Sociodemographic characteristics of students at Imam Abdulrahman Bin Faisal University (IAU), Eastern province, Saudi Arabia, 2023.

		No. (n = 2644)	%
College	Medicine	1127	42.6
	Non-medical	1517	57.4
Age	Min–max Mean ± SD	17–35 21 ± 3.1	
Educational level	Undergraduate	2514	95.1
	Postgraduate	130	4.9
Sex	Male	951	36.0
	Female	1693	64.0
Marital status	Married	235	8.9
	Single	2391	90.4
	Divorced or Widowed	18	0.7
Financial status	Not enough	640	24.2
	Enough	1658	62.7
	More than enough	346	13.1
Academic performance (GPA)	(<3.5) Good	371	14.8
	(3.5–4.49) Very Good	1282	51.0
	(>4.49) Excellent	861	34.2

**Table 2:** Characteristics of premature hair graying (PHG) in students at IAU, Saudi Arabia, 2023.

		No. (n = 2644)	%
Noticed graying of hair	Yes	1035	39.1
	No	1609	60.9
		PHG (n = 1035)	
Number of gray hairs	<10	720	69.6
	10–100	246	23.8
	>100	69	6.7
Age of noticing graying of hair (years)	Before 25	933	90.1
	25–<30	61	5.9
	30+	41	4.0
Use of hair oils before the onset of hair graying	No	728	70.5
	Yes	305	29.5
Use of minoxidil before the onset of hair graying	Yes	134	13.0
	No	899	87.0
Perceived Stress Scale	Low	152	14.7
	Moderate	677	65.3
	High	207	20.0

**Table 3:** Factors associated with premature hair graying (PHG) among IAU students.

		PGH (<30 years)				
		Yes (n = 994, 37.6%)		No (n = 1650, 62.4%)		*p* Value
		No.	%	No.	%	
College	Medicine (n = 1127)	422	37.4	705	62.6	0.891
	Non-medical (n = 1517)	572	37.7	945	62.3	
Sex	Male (n = 951)	366	38.5	585	61.5	0.478
	Female (n = 1693)	628	37.1	1065	62.9	
Marital status	Married (n = 235)	91	38.7	144	61.3	0.175
	Single (n = 2391)	900	37.6	1491	62.4	
	Widowed or divorced (n = 18)	3	16.7	15	83.3	
Body mass index (BMI)	Underweight (n = 414)	136	32.9	278	67.1	<0.001
	Normal (n = 1401)	484	34.5	917	65.5	
	Overweight (n = 541)	245	45.3	296	54.7	
	Obese (n = 288)	129	44.8	159	55.2	
Financial Status	Not enough (n = 640)	242	37.8	398	62.2	0.833
	Enough (n = 1658)	627	37.8	1031	62.2	
	More than enough (n = 346)	125	36.1	221	63.9	
Vitamin deficiency	B12 (n = 67)	18	26.9	49	73.1	0.002
	D (n = 523)	207	39.6	316	60.4	
	B12 and D (n = 278)	122	43.9	156	56.1	
	No (n = 400)	126	31.5	274	68.5	
Iron deficiency	Yes (n = 737)	287	38.9	450	61.1	0.376
	No (n = 808)	297	36.8	511	63.2	
Calcium deficiency	Yes (n = 209)	74	35.4	135	64.6	0.918
	No (n = 883)	316	35.8	567	64.2	
Depression	Yes (n = 211)	91	43.1	120	56.9	0.083
	No (n = 2428)	901	37.1	1527	62.9	
Allergic rhinitis	Yes (n = 316)	138	43.7	178	56.3	0.017
	No (n = 2323)	854	36.8	1469	63.2	
Atopic dermatitis	Yes (n = 208)	75	36.1	133	63.9	0.635
	No (n = 2431)	917	37.7	1514	62.3	
Bronchial asthma	Yes (n = 179)	60	33.5	119	66.5	0.244
	No (n = 2460)	932	37.9	1528	62.1	
Coronary artery disease	Yes (n = 7)	3	42.9	4	57.1	0.773
	No (n = 2632)	989	37.6	1643	62.4	
Hypothyroidism	Yes (n = 52)	20	38.5	32	61.5	0.896
	No (n = 2587)	972	37.6	1615	62.4	
Family history of PHG (before the age of 25)	Yes (n = 718)	443	61.7	275	38.3	<0.001
	No (n = 1925)	550	28.6	1375	71.4	
Number of daily steps	<3000 steps (Lack) (n = 799)	333	41.7	466	58.3	<0.001
	3000–6000 steps (Low) (n = 1069)	425	39.8	644	60.2	
	6001–10,000 (Moderate) (n = 1069)	195	30.9	437	69.1	
	>10,000 steps (Recommended) (n = 632)	40	28.2	102	71.8	
Diet	Vegetarian (n = 80)	24	30.0	56	70.0	0.119
	Mostly meat (n = 713)	286	40.1	427	59.9	
	Mixed meat and vegetables (n = 1850)	683	36.9	1167	63.1	
Smoking	Current smoker (n = 178)	86	48.3	92	51.7	0.037
	Past smoker (n = 54)	22	40.7	32	59.3	
	Secondhand smoker (n = 261)	97	37.2	164	62.8	
	Occasional smoker (n = 119)	47	39.5	72	60.5	
	Non-smoker (n = 2031)	741	36.5	1290	63.5	
Number of cigarettes ever smoked	100 + (n = 177)	92	52.0	85	48.0	0.003
	<100 (n = 170)	61	35.9	109	64.1	

**Table 4:** Logistic regression for independent factors associated with PGH.

	B	S.E.	Wald	Df	Sig.	Exp (B)	95% C.I. for Exp (B)	
							Lower	Upper
Medical college	0.006	0.087	0.005	1	0.946	1.006	0.848	1.194
Family history of PGH	1.434	0.094	231.163	1	0.000	4.197	3.489	5.050
Number of daily steps [24] (>10,000 steps)			22.800	3	0.000			
<3000 steps (Lack)	0.593	0.213	7.794	1	0.005	1.810	1.193	2.745
3000–6000 steps (Low)	0.538	0.209	6.605	1	0.010	1.712	1.136	2.580
6001–10,000 (Moderate)	0.124	0.218	0.325	1	0.569	1.132	0.738	1.737
Atopic dermatitis	−0.261	0.164	2.545	1	0.111	0.770	0.559	1.061
Bronchial asthma	−0.389	0.179	4.710	1	0.030	0.678	0.477	0.963
Allergic rhinitis	0.243	0.132	3.394	1	0.065	1.275	0.985	1.652
Depression	0.128	0.157	0.664	1	0.415	1.136	0.836	1.546
Coronary artery disease	0.104	0.802	0.017	1	0.897	1.110	0.231	5.338
Hypothyroidism	−0.121	0.313	0.150	1	0.698	0.886	0.480	1.635
Non-smoker			13.644	4	0.009			
Current smoker	0.602	0.168	12.900	1	0.000	1.826	1.315	2.537
Past smoker	0.280	0.302	0.858	1	0.354	1.323	0.732	2.389
Secondhand smoker	0.092	0.146	0.400	1	0.527	1.096	0.824	1.458
Occasional smoker	0.155	0.207	0.566	1	0.452	1.168	0.779	1.752
Underweight			32.903	3	0.000			
Normal	0.082	0.126	0.421	1	0.516	1.085	0.847	1.390
Overweight	0.604	0.145	17.333	1	0.000	1.829	1.377	2.431
Obese	0.552	0.168	10.747	1	0.001	1.737	1.249	2.416
Mixed meat and vegetables [8]			4.240	2	0.120			
Vegetarian	−0.320	0.266	1.445	1	0.229	0.726	0.431	1.223
Mostly meat	0.151	0.098	2.379	1	0.123	1.163	0.960	1.410
Constant	−1.654	0.230	51.669	1	0.000	0.191		

## Data Availability

Data supporting the findings and conclusions are available upon request from the corresponding author.

## Abbreviations

PHG	Premature hair graying
IAU	Imam Abdulrahman Bin Faisal University
PSS	Perceived stress scale
BMI	Body mass index
IRB	Institutional review board
